# Effect of brevican deficiency on neuroplasticity mediating molecules

**DOI:** 10.1186/2193-1801-4-S1-P55

**Published:** 2015-06-12

**Authors:** Solveig Weigel, Juliane Meissner, Alexander Zharkovsky, Thomas Arendt, Steffen Rossner, Markus Morawski

**Affiliations:** Paul Flechsig Institute for Brain Research, University of Leipzig, Germany; Department of Pharmacology (Biomeedikum), University of Tartu, Estonia

**Keywords:** extracellular matrix, brevican deficiency, tissue inhibitor of matrix metallo proteinase

Extracellular matrix (ECM) forms the pericellular part of the tissue. Due to the ECM’s constitution it defines the biophysical and biochemical properties of the tissue. In vertebrates’ brain the neuronal ECM mainly consists of negatively charged chondroitin sulfate proteoglycans (CSPG), hyaluronan and glycoproteins. CSPG are supposed to be involved in cell adhesion, axonal pathfinding and receptor binding. Therefore they are considered to play a role in neural development and plasticity as well as in several neurological and psychiatric disorders (e.g. schizophrenia and depression). One member of the CSPG family is brevican which was previously demonstrated by Blosa et al. (2013) to occur adjacent to the active zone of synapses. Accessorily, deficiency in brevican was reported to lead to a reduction of hippocampal LTP and recruitment of local plasticity. In this study we therefore focused on the effect of brevican on the neuronal cell adhesion molecule NCAM and its polysialylated form PSA-NCAM. Both of them are known to be involved in the establishment and modulation of neuroplasticity which are essential for memory formation. Furthermore we analysed the expression of the prolyl endopeptidase PREP, the matrix metalloproteinase 9 (MMP9) and tissue inhibitors of MMPs (TIMP). Immunohistochemical and western blot analyses of hippocampus tissue of brevican knockout mice compared with wild type littermates did not reveal changes in the expression of MMP9, PREP, NCAM nor PSA-NCAM, but showed a reduction of TIMP1 and 3 by trend. Consequently, changes of LTP and local plasticity in brevican deficient mice do not seem be mediated by an alteration of NCAM and PSA-NCAM, but might be supported by different expression levels of TIMP 1 and 3 modulating several MMPs’ activity.

